# Utilisation of ANC services before and after the COVID-19 pandemic in selected resource-poor blocks of India: role of community health workers in Swabhimaan programme area

**DOI:** 10.1186/s12913-023-09781-1

**Published:** 2023-08-14

**Authors:** Preeti Dhillon, Sayeed Unisa, Ajay Gupta, Abhishek Saraswat, Sulaiman KM, Sarang Pedgaonkar

**Affiliations:** 1 Department of Survey Research & Data Analystics, International Institute for Population Sciences, Mumbai, India; 2 Department of Bio-Statistics and Epidemiology, International Institute for Population Sciences, Mumbai, India; 3 Swabhimaan Project, International Institute for Population Sciences, Mumbai, India; 4 Department of Family & Generations, International Institute for Population Sciences, Mumbai, India

**Keywords:** COVID-19, Antenatal Care, Community Health Worker

## Abstract

**Introduction:**

COVID-19 has disrupted maternal and child health services. Community Health Workers (CHWs) supported the women by visiting pregnant women's homes and providing the MCH services as required. This study attempts to understand the role of CHW and its impact on the Ante-Natal Care (ANC) services pre-pandemic and post-Pandemic in the poor resource setting.

**Methods:**

The *Swabhimaan* programme interventions were carried out in the selected blocks in the Indian States of Bihar, Odisha and Chhattisgarh with the objective to improve the nutritional status of mothers, pregnant women and adolescents living in resource-poor blocks of three selected states during 2016–2022. Cross-sectional surveys, namely pre-pandemic (2018–19) and post-pandemic (2021–22) of pregnant and mothers of under two children, utilised to fulfil the objectives of this study. These surveys are part of *Swabhimaan* evaluation, a community-based non-randomised controlled study.

**Results:**

The ANC services received by women have increased over time from 2015 to 2022. Our findings confirm that the ground-level community and health systems were active during the pandemic, and the results show significant improvement. Additionally, the women supported by the CHW have substantially improved pregnancy registration, first ANC, Tetanus injection, consumption of Iron Folic Acid, Calcium and deworming tablets than those who did not. Propesnsity Score Matching analysis shows that the average treatment effect on the various ANC services of having the support of CHW is significant.

**Conclusion:**

This study shows the vital role of CHWs in utilising various Maternal and Child Health services. Better linkage and networking of the CHWs with the community will ensure health service delivery regularly and in an emergency like a pandemic and develop resilience.

## Introduction

The COVID-19 pandemic posed unprecedented challenges by straining the under-resourced health systems in low- and middle-income countries (LMICs) [1]. It affected the workforce and supplies of, demand for, and access to health systems and worsened mortality and morbidity due to changes in care priority [2]. Disruptions in maternal health services have been a significant concern since the COVID-19 pandemic began in early 2020. Experts predicted that such disruptions might lead to a 10% decline in the utilisation of reproductive and maternal health services in LMICs [3]. In a survey conducted by the WHO, 53% of the 105 participating countries reported partial disruptions in antenatal care (ANC) and 32% disruptions in institutional delivery services during the first few months of the COVID-19 pandemic [4].

Pregnant women are a vulnerable group who need special consideration during any health emergency [5]. During the earlier epidemics (Ebola and SARS), regular healthcare services [6, 7], mainly the essential healthcare services for pregnant women, were disturbed and led to higher maternal mortality [8]. Previous studies have documented numerous sociocultural, geographical and economic barriers for women in LMICs to access maternal health services, including lack of transportation, long distance to a reliable healthcare facility, limited skilled workers in rural areas, and financial barriers [9]. The COVID-19 pandemic was feared to aggravate these barriers for people who were more exposed than the rest to the economic difficulties associated with the pandemic [10].

India announced a nationwide lockdown on 24 March 2020, which continued for more than six months and was later extended with a few relaxations here and there. Despite the mitigation measures, India was at the top among the LMICs regarding the number of confirmed COVID-19 cases [11]. Forced by the weakness of their public health systems, LMICs including India diverted their resources to manage the outbreak of COVID-19, disrupting essential health services, including maternal healthcare services [12]. It is critical to assess the severity of such disruptions to alleviate their impact on health outcomes [13]; particularly in resource-poor rural areas where the swabhimaan programme was active to strengthen the existing health and nutrition delivery system and ensure community participation. The *Swabhimaan* is an initiative of UNICEF India in collaboration with the National Rural Livelihoods Mission (NRLM), Government of India. It was launched in 2016 to improve the nutritional status of adolescent girls, pregnant women, and mothers of children under two years in the selected resource-poor blocks of three Indian states, namely Odisha, Chhattisgarh, and Bihar [14].

Given the nature of COVID-19, literature that scientifically quantifies the implications of the pandemic on the use of maternal health care in LMICs is limited [15]. One of the major drawbacks is the lack of reliable data to determine significant differences in the use of services before and after the outbreak of COVID-19 [16–18]. A limited available research in Indian context reveals that the COVID-19 pandemic reduced the utilisation of various antenatal health care services among pregnant women as compared to the pre-pandemic period [19]. Therefore, It is imperative to expand the current literature; therefore, this study examines the role and effectiveness of the existing community health workers' services in ensuring maternal healthcare services during the COVID-19 health emergency.

To achieve Sustanable Development Goals even at local level, the health workers from the communities play a vital role in providing ground level services. ASHA (Accredited Social Health Activist), ANM (Auxiliary nurse midwife), and AWW (Anganwadi worker) are a part of the continuum of care for women and play a crucial role in it. ASHA, generally a woman, is an interface between the community and the public health system and is accountable for ensuring the smooth implementation of the National Health Mission (NHM) at the lowest level, that is, village or slum pocket. ANMs are a crucial component of NHM, connecting ASHAs and communities with the public health system and delivering specific services through camps, home visits, and PHCs. As community members, Anganwadi workers (AWWs) assist ANMs and ASHAs provide services, executing programs, and maintaining records under the Integrated Child Development Scheme (ICDS). The government of India implemented a lockdown to curtail the spread of COVID-19, with sealed boundaries and restrictions of movement. Only local-level community health workers could provide ANC services to pregnant women during this situation. Therefore, this study assesses the role of community health worker during the pandemic by using *Swabhimaan* midline and endline impact evaluation surveys.

## Material and methods

### Study setting

The *Swabhimaan* programme has three cross-sectional surveys: baseline (2016–17), midline (2018–19), and endline (2021–22). During all three rounds, data was collected from the Kasba and Jalalgarh blocks in the Purnea district of Bihar, the Pallahara (Angul district) and Koraput (Koraput district) blocks of Odisha, and the Bastar and Bakawand blocks in the Bastar district of Chhattisgarh. The present study evaluates the data from the midline survey carried out before the pandemic in 2018–19 (Sep’18-Jun’19) and the endline survey conducted after the onset of COVID-19 during 2021–22 (Jan’21-Jan’22).

### Study design and sampling

In designing this cross-sectional study, we took into account the Swabhimaan strategy and the outcome indicators, as well as the changes anticipated. The sample selection was based on two-stage systematic sampling and was designed to provide the estimates of key indicators at block levels across the three selected states. The representative sample in each round was derived using the simple random sampling after adjusting for non-response rate and the required women were selected from sampling frame using the sysytematic sampling. The detailed information on survey design of this prospective, non-randomised controlled study is available elsewhere [14]. The number of pregnant women (PW) and mothers of children under two (MU2) interviewed during the two rounds is shown in Table 1. For this study, pregnant women in the second and third trimesters were considered as most registered or visited health facilities for check-ups before the second trimester and started utilising the pre-natal services.

**Table 1:** Pregnant Women (PW-in second and third trimester) and Mothers of children under two years (MU2) interviewed pre-pandemic (2018–19) and post-pandemic(2021–22) in Swabhimaan Intervention Area

State	**Pre-Pandemic (2018–19)**		**Post-Pandemic (2021–22)**	
	**PW**	**MU2**	**PW**	**MU2**
Bihar	520	1042	370	1162
Odisha	458	1183	460	1522
Chhattisgarh	439	1051	621	2082
	**1417**	**3276**	**1450**	**4766**

### Tools

In all rounds of surveys, trained field investigators administered pretested bilingual questionnaires to pregnant and MU2 women through face-to face computer-assisted personal interviews (CAPI). This data collection application used in the CAPI ensures quality data collection in timely manner with inbuilt soft and hard checks. Further, the quality control checks on several issues including ANC services were done for 10 percent of the sampled women in every state and in each round to maintain the quality of the data.

### Variables

#### Outcome variables

The variables representing maternal health care services were pregnancy registration, seeking ANC in the first trimester, and receipt of Tetanus (TT) injection, Iron Folic Acid (IFA) tablets, calcium tablets, and deworming tablets in the second and third trimesters of pregnancy.

#### Exposure variables

Community workers providing advice about ANC services, helping with early pregnancy registration, counselling on birth preparedness, and supporting ANC services during COVID-19 were treated as independent variables. Other covariates, such as state, age, education, religion, caste, family size, and return of migrant members during COVID-19, were included to understand the differentials. The return of a migrant member was considered a possible covariate as households with such members may have been left out by community health workers (CHWs) due to COVID-19, which may have impacted mothers or pregnant women in those households.

### Statistical approach

The utilisation of ANC services for pregnant women of second and third trimesters is examined. In the case of mothers under two years, these services are completed; hence, there are no exclusion criteria for the analysis. The trend of ANC service utilisation is based on three rounds of surveys, to show the impact of intervention. Authors analysed the involvement of community health workers like ASHAs, ANMs, and AWWs in creating awareness regarding ANC, providing counselling, and supporting women while seeking ANC during the pre and post-pandemic periods separately for PW and MU2(2018–19 and 2021–2022). ANOVA is used to understand the differentials in exposure and outcome variables by womens varying characteristics post-covidLastly, the role of the CHWs in utilising ANC by women was explored using bivariate analysis, and the impact of the services provided by the CHWs was analysed using the propensity score method (PSM). The matching technique was used because the beneficiaries were not randomly selected, as a result of which selection bias could be introduced in the estimated impact of the programme. The covariates matched in the present study encompassed categorical variables like caste, education, religion, type of house, and availability of land. The analysis was carried out in the following steps.Firstly, the logit model was used to estimate the propensity score for the covariates presenting mothers' demographic and socio-economic characteristics. Next, we used the k-match command to calculate the difference, known as the average treatment effect (ATE), in the utilisation of maternal health services between the control group (those who didn't receive or avail services through CHWs) and the treatment group (those who received assistance from the CHWs). The difference is presented in terms of proportion.

There are basic assumptions that need to be satisfied for using PSM. The first is that the potential outcomes must be determined in a way that they are independent of the treatment assignment, possibly carried out by including all the covariates that affect the outcome or the treatment. The second is a common support or overlap condition; that is, the treatment units will have to be similar to control units in observed characteristics unaffected by the participation of CHWs. The data was collected using CS Pro (Census and survey processing system) and extracted and analysed using STATA 15.1.

## Results

In Bihar and Odisha, an endline survey was conducted after the first wave of COVID-19. In the state of Chhattisgarh, the survey was conducted after the second wave of COVID-19. Most of the women in both groups are below 30 years (Table 2). The educational situation of women is bad in the study area as more than one-third of all the women (both pregnant and mothers of children under two) had never attended school. Pregnant women and mothers of children under two mostly belonged to Hindu households. More than sixty percent of the sampled pregnant women and mothers of children under two belonged to the scheduled tribes and scheduled caste. Around 5 in 10 pregnant women lived in a family of less than five members, while a similar proportion of mothers of children under two lived in a family of more than four members during the endline survey. Around 40 percent of the women reported that their household experienced return migration of members during the first wave of COVID-19, March-June 2022.

**Table 2:** Background characteristics of pregnant women (second & third trimesters) and mothers of children under two years in the Swabhimaan survey area

	**Pregnant Women**^**a**^		**Mothers**	
	**Pre-pandemic (2018–19)**	**Post-pandemic (2021–22)**	**Pre-pandemic (2018–19)**	**Post-pandemic (2021–22)**
**State**				
Bihar	36.7	25.5	31.8	24.4
Odisha	32.3	31.7	36.1	31.9
Chhattisgarh	31.0	42.8	32.1	43.7
**Age group**				
15–19	13.5	12.5	7.5	6.8
20–29	71.8	69.0	72.4	70.3
30 and above	14.7	18.5	20.1	22.9
**Education Status**				
Never attended	41.8	32.7	43.9	35.9
1–9 Years	41.2	38.7	39.2	39.3
10 Years and more	16.9	28.6	16.8	24.8
**Religion**				
Hindu	61.8	83.4	82.1	83.3
Others	38.2	16.6	17.9	16.7
**Caste**				
Scheduled Caste	15.7	21.7	14.4	23.4
Scheduled Tribe	38.5	39.7	41.8	40.5
Other Backward Castes	24.5	28.4	24.8	25.4
Others	21.4	10.3	19.0	10.7
**Family Size**				
1–4 members	53.6	61.2	37.2	41.8
5 and more	46.4	38.8	62.8	58.2
**Any member of the household returned during the COVID-19**				
No	-	60.7	-	61.2
Yes	-	39.3	-	38.8
**Total Sample**	**1417**	**1450**	**3276**	**4766**

A: Pregnant Women who were in second and third trimesters

**Fig. 1:**
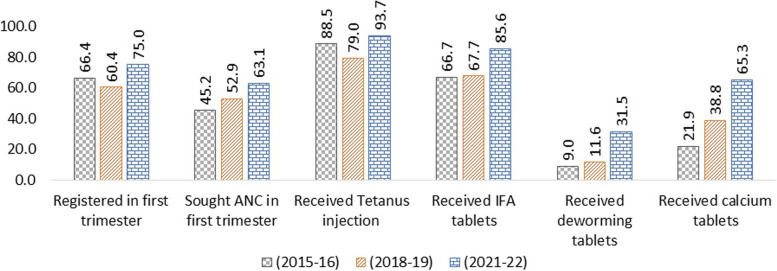
Percentage of pregnant women who received various ANC services over time

**Fig. 2:**
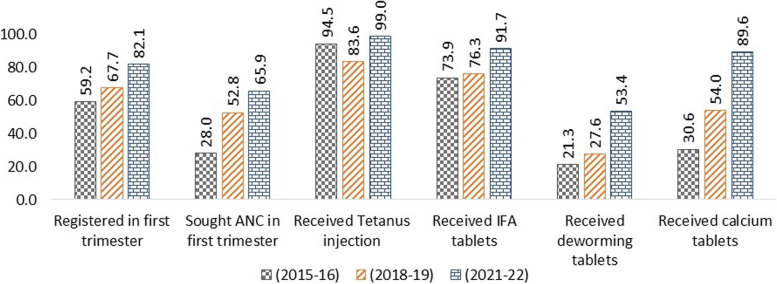
Percentage of mothers who received various ANC services during pregnancy over time

In the Swabhimaan area, essential ANC indicators have improved from 2015 to 2022 for pregnant women and mothers of under two children (Figs. 1 and 2). The increase in the use of IFA tables, Tetanus injection and calcium tables is impressive. Not a single indicator has shown a decrease despite the pandemic. Mothers of under two children must have been pregnant during the pandemic, and their ANC utilisation has also demonstrated an impressive increase.

ASHAs, ANMs, and AWWs played a significant role during COVID-19 in three states. Around 60 percent (Table 3) of the women (PW and MU2) received advice on ANC services from ASHAs before COVID-19, while more than three-fourths did so after COVID-19. More than 87 percent of the women were advised by different CHW taken together after COVID-19 as compared to the pre-COVID period (PW: 72 percent, MU2: 76.9 percent).

**Table 3:** Percentage of women who received advice and counselling support for ANC services from community health workers during the pre-and post-COVID periods

		**Pregnant Women**^**a**^			**Mothers of Children under two**		
		**Pre-COVID (2018–19)**	**Post-COVID (2021–22)**	**Significant Diff (pre-post)**	**Pre-COVID (2018–19)**	**Post-COVID (2021–22)**	**Significant Diff (pre-post)**
**Advising about ANC services**	ASHA	61.8	77.5	***	65.4	80.5	***
	ANM	36.5	62.9	***	38.2	68.5	***
	AWW	42.1	72.4	***	46.6	77.5	***
	**At least one of them **^**Q**^	72.0	87.4	***	76.9	89.9	***
**Awareness about early pregnancy registration**	ASHA	60.3	83.9	***	61.9	82.8	***
	ANM	20.7	53.1	***	22.0	58.1	***
	AWW	36.7	70.2	***	39.1	77.4	***
	**At least one of them **^**Q**^	71.5	92.3	***	75.0	94.1	***
**Help with pregnancy registration**	ASHA	8.7	61.9	***	67.4	78.7	***
	ANM	37.6	34.0	***	27.8	55.1	***
	AWW	53.8	55.1		47.5	75.3	***
	**At least one of them **^**Q**^	73.5	75.2		87.6	95.2	***
**Counselling on birth preparedness**	ASHA	47.6	63.2	***	65.0	84.3	***
	ANM	25.8	54.2	***	32.6	68.0	***
	AWW	28.1	57.2	***	38.7	77.7	***
	**At least one of them **^**Q**^	53.4	70.7	***	71.9	91.8	***
**ANC Support During COVID-19**	ASHA	-	66.1		-	35.6	
	ANM	-	49.8		-	28.7	
	AWW	-	51.0		-	30.8	
	**At least one of them **^**Q**^	-	72.3		-	39.0	
Total Sample		**1417**	**1450**		**3276**	**4766**	

^#^ Pre-COVID: Midline survey of Swabhimaan; Post-COVID: Endline survey of Swabhimaan

^***^ depicts the signicant differences between the pre- and post-covid (Proportion test); Diff.-Difference

A: Pregnant women who were in second and third trimesters

Q: At least one of them (ASHAs, ANMs, or AWWs)

The results also show a significant increase in the number of women who got help from an ASHA in pregnancy registration in the post-COVID period (Table 2: PW: 8.7% to 62%; MU2: 67.4% to 78.7%). There was a minor increase in the proportion of currently pregnant women who received help from all CHWs put together (73.5 percent pre-COVID to 75.2 percent post-COVID). The reach of ASHAs, ANMs, and AWWs in informing women of the importance of early pregnancy registration and in providing birth counselling increased post-COVID. More than 50 percent of the pregnant women and one-fourth of the mothers reported receiving support for ANC services from all three CHWs during COVID-19 in these states.

Furthermore, it examined the involvement of CHWs during pre and post-pandemic by characteristics of pregnant and MU2 women. Help and counselling received by all the women may not be similar and differs by the background charactersistics. So, Using the bivariate methods, it was found that there was a significant difference in the prevalence of advice, help, and counselling received pre- and post-COVID by the indivuals characteristics. In the states of Odisha and Chhattisgarh, more than three-fourths of the currently pregnant women received support for ANC services and help with pregnancy registration from at least one CHW during COVID-19 (Table 4). In contrast, around 60 percent of currently pregnant women in Bihar received support for ANC services, help with pregnancy registration and counselling on birth preparedness from at least one of the CHWs during COVID-19 (Table 4). More women with higher education reported receiving ANC support, help with pregnancy registration, and counselling services from any CHWs during COVID-19 compared to women with less than 10 years of schooling. However, the differential in proportion was not very much. However, all PW received support from at least one of the community health workers, irrespective of their level of education. A more significant number of SC/ST women than their counterparts and more than 70 percent of Hindu PW received support, help, and counselling from any CHWs. A slightly higher number of PW living in households with return migrant members received ANC support from ASHA, ANM, and AWW. More than 85 percent of the mothers got help with pregnancy registration and counselling on birth preparedness from CHWs. Less than 50 percent of women (MU2) received support for ANC services, irrespective of their background characteristics. The reach of ASHA in supporting women for ANC services, helping with pregnancy registration, and counselling on birth preparedness was comparatively more than that of ANM and AWW for both PW and mothers.

**Table 4:** Percentage of women who received help, counselling, and support from community health workers post-COVID by women's background characteristics

Pregnant Women (Second/ Third Trimester)												
	**Received ANC Support during COVID-19**				**Help with pregnancy registration**				**Counselling on birth preparedness**			
	**ASHA**	**ANM**	**AWW**	**At least one of them **^**Q**^	**ASHA**	**ANM**	**AWW**	**At least one of them **^**Q**^	**ASHA**	**ANM**	**AWW**	**At least one of them **^**Q**^
	**Yes**	**Yes**	**Yes**	**Yes**	**Yes**	**Yes**	**Yes**	**Yes**	**Yes**	**Yes**	**Yes**	**Yes**
**State**	***	***	***	***	***	***	***	***	***	***	***	***
Bihar	58.6	20.3	22.4	60.5	58.1	17.3	27.3	60	60.3	23.8	27.6	62.4
Odisha	70.6	58.8	59	75.4	67.8	32.7	65.8	87.1	57.7	55.3	58.2	63.2
Chhattisgarh	67.1	60.7	62.2	77.1	59.9	44.9	63.8	75.5	68.9	71.5	74.1	81.2
**Education Status**	***	***	***	*		*	***			**	**	
Never attended	65.8	47.3	47	71.5	61.2	30.2	49.8	72.2	62.2	48.9	51.7	68.6
1–9 Years	62.2	46	48.1	70.1	61.9	35.1	55.6	76.3	64.9	55.3	59	72.7
10 years and more	71.6	57.8	59.5	76.4	62.9	36.9	60.5	77.3	61.9	58.8	61	70.4
**Religion**		***	***	***	*	***	***	***	***	***	***	***
Hindu	66.7	54.8	55.8	73.9	62.6	36.6	59.7	77.9	63.4	59.6	62.9	72.1
Others	62.9	24.6	27.1	64.6	58.3	20.8	32.1	62.1	62.1	27.1	28.3	63.3
**Caste**	*	***	***	***	***	***	***	***	**	***	***	**
Scheduled Caste	72	58	59.2	79	57.6	41.1	59.6	76.8	67.5	61.8	63.4	74.8
Scheduled Tribe	65.2	52.3	54.1	72.3	67.7	31.1	59.1	79.1	59.5	55	59	68.2
Other Backward Castes	64.3	48.1	48.3	69.9	58.3	35.4	52.7	71.8	66.3	54.6	57	73.5
Others	61.7	27.5	29.5	65.1	59.1	26.2	36.9	66.4	59.7	34.2	37.6	63.8
**Family Size**		**										**
3–4 members	67.1	52.1	52.5	73.2	62	32.8	54.7	75.5	61.6	53.9	56.4	68.8
5 and more	64.4	46.1	48.8	71	61.7	35.9	55.7	74.9	65.7	54.6	58.4	73.7
**Any member of the household returned during the COVID-19**	**	***	***	**			***	***	*			
No	63.8	46.1	47.7	70.1	62.5	32.8	59.7	78.8	61.5	54.9	58.4	70.5
Yes	69.6	55.4	56.1	75.8	61.1	35.8	48.1	69.8	65.8	53.2	55.3	71.1
**Mothers of Children under two**												
	**Received ANC Support during COVID**				**Help with pregnancy registration**				**Counselling on birth preparedness**			
	**ASHA**	**ANM**	**AWW**	**At least one of them **^**Q**^	**ASHA**	**ANM**	**AWW**	**At least one of them **^**Q**^	**ASHA**	**ANM**	**AWW**	**At least one of them **^**Q**^
	**Yes**	**Yes**	**Yes**	**Yes**	**Yes**	**Yes**	**Yes**	**Yes**	**Yes**	**Yes**	**Yes**	**Yes**
**State**	***	***	***	***	***	***	***	***	***	***	***	***
Bihar	24.5	11.8	15.5	25.2	85.5	36.2	54	89.3	83.2	35.7	49.1	85.5
Odisha	39	32.3	33.6	41.5	75.6	52.6	78.8	97	82.7	76	82.9	90.2
Chhattisgarh	39.4	35.5	37.3	45	77.2	67.4	84.5	97.2	86.2	80.1	89.9	96.4
**Education Status**	**	***	***	**		**			*	***	***	***
Never attended	34.1	25.4	27.9	37.2	79.1	52.3	74.1	95	82.7	62.6	74.3	90.2
1–9 Years	34.8	29.1	31.1	38.5	79	56.4	75.5	95.6	85.5	69.7	79.6	92.6
10 Years and more	39.2	32.8	34.4	42.5	77.6	57	76.5	95	84.8	73.1	79.5	92.9
**Religion**	***	***	***	***	***	***	***	***		***	***	***
Hindu	37.7	32	33.7	41.6	77.8	58.6	79.1	96.4	84.7	73.5	82.5	93.1
Others	25.2	12.3	16.3	26.1	83.2	37.6	56.2	89.5	82.4	40.5	53.6	85.4
**Caste**	***	***	***	***	***	***	***	***		***	***	**
Scheduled Caste	42.2	38.2	38	45.9	73.7	63.2	77.6	95.2	85.1	75.3	82.7	93.5
Scheduled Tribe	36	28.5	31.9	39.7	78.7	54.4	80.6	96.3	84.2	71.7	83.1	92.2
Other Backward Castes	33.1	25.8	28.6	36.3	81.3	51.9	70.9	94.5	83.2	64.4	72.8	90.4
Others	25.8	15.5	16.2	28.2	83.8	47.7	60.3	93	86.1	46.4	57.7	89.6
**Family Size**				*		***	*	**				**
3–4 members	35.1	28	29.9	37.5	79	52	74	94.4	83.5	67.3	76.5	90.8
5 and more	36	29.2	31.4	40.2	78.6	57.3	76.1	95.8	85	68.5	78.5	92.5
**Any member of the household returned during the COVID-19**	***	***	***		***	***		**	***	**		**
No	34.5	26	29.2	39	76.7	50.7	75	95.9	81.8	66.8	77.3	91.1
Yes	37.3	32.8	33.3	39.2	81.9	62	75.6	94.2	88.3	69.9	78.2	92.9

^*^ *P*-value < 0.10, **p-value < 0.05, ***p-value < 0.01 depicts the significant differences between the set of variables (ANOVA); # Endline, a-Pregnant women who were in second and third trimesters; Q: At least one of them (ASHA, ANM, or AWW)

More women from the state of Odisha than those from the other two states reported registering their pregnancy and utilising at least one ANC service in the first trimester (Table 5). Likewise, more PW and MU2 received calcium tablets in Odisha than in the other two states. Compared to women with less than 10 years of education, a higher proportion of those with at least 10 years had utilised any ANC services, be it registering their pregnancy in the first trimester or receiving TT injection or IFA or calcium tablets. Hindu women or SC/ST women were ahead of their counterparts in utilising antenatal services. PW and MU2 who had less than five members in their household or with return migrant members during COVID-19 were more to utilise the pre-natal services than their counterparts.

**Table 5:** Percentage of women who received various ANC services by their background characteristics post-COVID

	**Registered in the first trimester**	**Sought ANC in the first trimester**	**Received Tetanus injection**	**Received IFA tablets**	**Received deworming tablets**	**Received calcium tablets**
**Pregnant women**^a^						
	**Yes**	**Yes**	**Yes**	**Yes**	**Yes**	**Yes**
**State**	***	***	***	***	***	***
Bihar	51.1	45.7	87.6	74.6	17.6	58.1
Odisha	90.0	71.0	96.5	86.9	35.3	65.4
Chhattisgarh	78.3	67.6	95.2	91.1	37.0	69.6
**Education Status**	***	***	***	***		
Never attended	69.0	55.9	90.1	82.1	31.2	62.4
1–9 Years	76.1	62.9	95.4	84.5	32.1	64.9
10 years and more	80.5	71.6	95.4	91.1	31.1	69.2
**Religion**	***	***	***	***	***	*
Hindu	80.2	67.0	94.5	87.4	34.4	66.4
Others	49.2	43.3	89.2	76.3	17.1	60.0
**Caste**	***	**	*	***	***	***
Scheduled Caste	76.1	67.8	95.2	88.2	39.2	71.7
Scheduled Tribe	81.4	64.7	94.6	86.8	30.8	64.5
Other Backward Castes	68.4	58.0	92.7	86.2	31.6	66.0
Others	66.4	61.1	89.3	73.8	18.1	53.0
**Family Size**	**	***				
3–4	77.1	65.8	93.8	86.3	30.5	64.0
5 and more	71.7	58.9	93.4	84.5	33.1	67.4
**Any member of the household returned during the COVID-19**	***	***	***	***	**	***
No	79.7	69.0	96.7	88.5	33.5	68.2
Yes	67.9	54.0	88.9	81.1	28.4	60.9
**Mother**						
	**Yes**	**Yes**	**Yes**	**Yes**	**Yes**	**Yes**
**State**	***	***	***	***	***	***
Bihar	60.8	50.6	99.1	88.8	41.7	84.3
Odisha	91.3	73.8	98.0	92.8	69.1	94.7
Chhattisgarh	87.2	68.8	99.7	92.5	48.6	88.8
**Education Status**	***	***			*	***
Never attended	77.3	59.1	99.2	91.2	53.1	87.1
1–9 Years	82.5	67.6	98.9	91.8	51.9	90.3
10 years and more	88.4	73.3	98.9	92.1	56.4	92.0
**Religion**	***	***		***	***	***
Hindu	86.7	69.2	99.0	92.5	55.2	90.7
Others	59.0	49.6	99.0	87.5	44.5	83.9
**Caste**	***	***	**	*	***	***
Scheduled Caste	83.1	69.0	99.5	92.8	60.5	89.9
Scheduled Tribe	86.6	67.7	98.8	91.5	53.7	91.0
Other Backward Castes	76.8	63.0	98.7	92.1	50.5	90.2
Others	75.3	59.7	99.8	88.8	44.2	82.4
**Family Size**	**	**				**
3–4	83.5	67.7	98.8	91.6	53.0	90.9
5 and more	81.0	64.7	99.2	91.7	53.8	88.6
**Any member of the household returned during the COVID-19**	***	***	***	**		
No	84.2	68.2	99.3	92.4	53.1	89.2
Yes	78.7	62.4	98.5	90.5	54.0	90.2

^*^P-value < 0.10, **p-value < 0.05, ***p-value < 0.01 depicts the significant differences between the set of variables (ANOVA); # Endline, a-Pregnant women who were in second and third trimesters

It was found that pregnant women and mothers who had received ANC support and help with pregnancy registration from an ASHA/any other CHW during COVID-19 had also utilised antenatal services compared to those who had not received such support (Table 6). More than 90 percent of pregnant women who had received support for ANC services also received IFA tablets compared to women without such support. A significant difference was observed in the consumption of deworming tablets among mothers and pregnant women who had received ANC support and help with pregnancy registration from ASHA or any CHW during COVID-19 compared to those who had not received ANC support. Double the number of mothers who had gotten help with pregnancy registration from ASHA or any other CHW received ANC in the first trimester than those who had not received support from a CHW.

**Table 6:** Percentage of women who received various ANC services by the support received from community health workers post-COVID

Help and Support from Community Health Workers			Registered in the first trimester	Sought ANC in the first trimester	Received Tetanus injection	Received IFA tablets	Received deworming tablets	Received calcium tablets
**Pregnant Women**^**a**^								
Received ANC Support during COVID	ASHA ***	No	66.3	57.5	89.8	75.4	21.1	52.6
		Yes	79.5	66.0	95.6	90.8	36.8	71.8
	At least one of them ^Q^ ***	No	64.6	57.4	89.5	72.6	19.5	51.4
		Yes	79.0	65.3	95.2	90.6	36.1	70.6
Received help with pregnancy registration	ASHA ***	No	56.0	53.3	87.7	81.7	27.4	51.3
		Yes	86.7	69.2	97.3	88.0	34.1	73.9
	At least one of them ^Q^ ***	No	37.9	46.8	83.8	77.7	20.9	39.6
		Yes	87.3	68.5	96.9	88.2	35.0	73.8
**Mother of Children under two**								
Received ANC Support during COVID	ASHA ***	No	80.4	65.7	98.7	88.3	40.5	85.9
		Yes	85.1	66.3	99.6	97.8	76.7	96.3
	At least one of them ^Q^ ***	No	80.0	65.8	98.6	87.7	38.6	85.3
		Yes	85.4	66.2	99.6	97.9	76.6	96.2
Received help with pregnancy registration	ASHA ***	No	72.3	52.9	97.5	89.8	54.5	85.6
		Yes	84.7	69.5	99.4	92.2	53.1	90.7
	At least one of them ^Q^ ***	No	20.2	34.2	91.7	78.1	41.2	72.8
		Yes	85.2	67.5	99.4	92.4	54.1	90.4

^*^ *P*-value < 0.10, **p-value < 0.05, ***p-value < 0.01 depicts the significant differences within the set of variables (t-test); # Endline, a: Pregnant women who were in second and third trimesters

Q: At least one of them (ASHAs, ANMs, or AWWs)

**Fig. 3:**
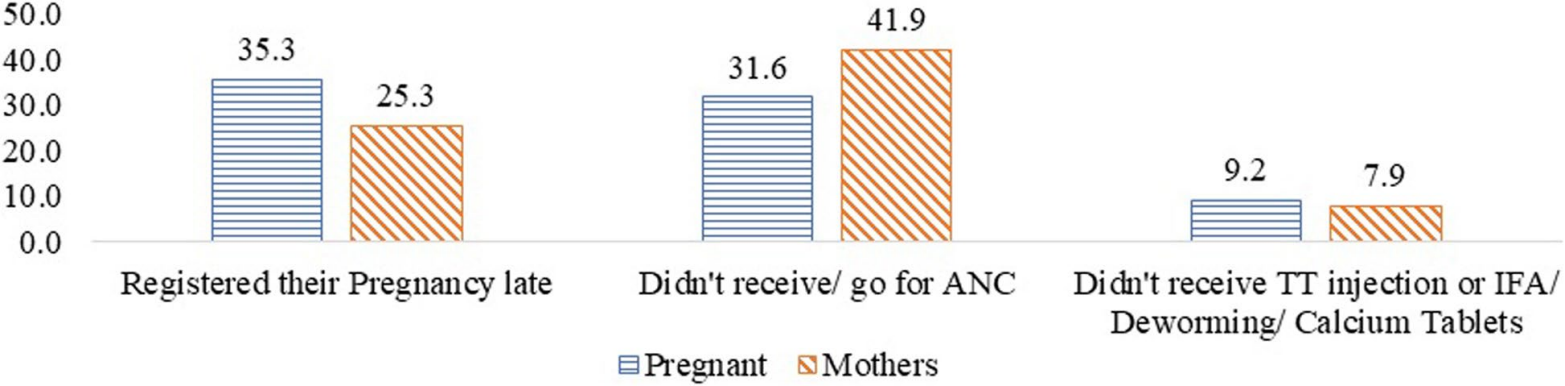
Percentage of Women who reported COVID-19 pandemic as the reason for not availing ANC Services. Note: a. Women who had registered for ANC registered their pregnancy late. b. Women who did not receive/ go for ANC among all women respondents. c. Women who did not receive/avail of at least one of the services (TT/IFA/Calcium or Deworming tablets) among those who had registered their pregnancy

More than 35 percent of currently pregnant women and one-fourth of mothers reported COVID-19 as one of the reasons for their late pregnancy registration (Fig. 3). Thirty-one percent of the currently pregnant women and 42 percent of the mothers didn't avail of ANC services due to the COVID-19 pandemic. Also, more than 8 percent of women reported not receiving TT injections or IFA/Deworming/Calcium tablets due to the pandemic.

The results from the Table 4 and Table 5 clearly shows the significants difference by the background characteristcis in exposure and outcome variables. Hence thers is possibility of examining the role of community health workers using the preonsperity score mataching method. Table 7 demonstrates that pregnant women received the most ANC support from ASHA workers during the pandemic. The impact of ASHA was high on pregnancy registration, receipt of first ANC, and receipt of calcium tablets among pregnant women during the pandemic compared to other CHW help in pregnancy registration. The treatment effect of CHWs was highest on the overall pregnancy registration by the women (0.45 for PW and 0.39 for MU2). It seems that the CHWs' role is not impacting the receipt of TT injections for either of the target groups.

**Table 7:** Average treatment effect (ATE) of help and support from community health workers on ANC services received by women post-COVID

Help and support from community health workers		Registered in the first trimester	Sought ANC in the first trimester	Received Tetanus injection	Received IFA tablets	Received deworming tablets	Received calcium tablets
**Pregnant women**^**a**^							
Received ANC support during COVID-19	ASHA	0.12 ***	0.07 ***	0.06 ***	0.14 ***	0.14 ***	0.19 ***
		(0.02)	(0.03)	(0.02)	(0.02)	(0.03)	(0.03)
	**At least one of them **^**Q**^	0.13 ***	0.06 **	0.07 ***	0.17 ***	0.16 ***	0.19 ***
		(0.03)	(0.03)	(0.02)	(0.03)	(0.03)	(0.03)
Received help with pregnancy registration	ASHA	0.29 ***	0.15 ***	0.1 ***	0.06 ***	0.07 ***	0.23 ***
		(0.02)	(0.03)	(0.01)	(0.02)	(0.02)	(0.03)
	**At least one of them **^**Q**^	0.45 ***	0.17 ***	0.11 ***	0.07 ***	0.13 ***	0.33 ***
		(0.03)	(0.03)	(0.02)	(0.02)	(0.03)	(0.03)
**Mothers of children under two**							
Received ANC support during COVID-19	ASHA	0.03 ***	0	0.01 ***	0.1 ***	0.35 ***	0.1 ***
		(0.01)	(0.01)	(0)	(0.01)	(0.01)	(0.01)
	**At least one of them **^**Q**^	0.04 ***	-0.01	0.01 ***	0.1 ***	0.37 ***	0.1 ***
		(0.01)	(0.01)	(0)	(0.01)	(0.01)	(0.01)
Received help with pregnancy registration	ASHA	0.16 ***	0.19 ***	0.02 ***	0.04 ***	-0.01	0.06 ***
		(0.02)	(0.02)	(0.01)	(0.01)	(0.02)	(0.01)
	**At least one of them **^**Q**^	0.39 ***	0.25 ***	0.04 ***	0.1 ***	0.05 *	0.1 ***
		(0.03)	(0.03)	(0.01)	(0.02)	(0.03)	(0.02)

^*^P-value < 0.10, **p-value < 0.05, ***p-value < 0.01 depicts the significant ATE

^a^Endline, a: Pregnant women who were in second and third trimesters

Q: At least one of them (ASHAs, ANMs, or AWWs)

(..)-Standard Deviation

## Discussion

The findings of this study reveal that in the study area, despite the pandemic, the proportion of pregnant women utilising various components of ANC increased from pre-pandemic (2019) to post-pandemic (2022) After the pandemic's breakout, pregnancy registration in the first trimester increased by 15 percent, micronutrient supplementation like IFA recorded an about 20 percent increase, and compliance with deworming tablets increased from 11 to 31 percent. Corroborating our findings, Nguyen et al. elaborated that although fewer health facilities provided ANC services amid the lockdown, most services for pregnant women continued to be offered, except for anthropometric measurements, which registered a decline [20]. The restrictions during the pandemic resulted in a drop in the use of essential maternal care services like weight monitoring, IFA and calcium supplementation, and counselling [21]. The impressive contribution of CHWs in the study area is revealed in the present study. This trend is expected as due to a lower coverage of ANC at baseline, and therefore the blocks were targeted by programmes including *swabhimaan* intervention included engaging the community resource members, self help group members to closely work with the CHWs to continue the services that pregnant women and new mothers should receive. The intervention focuses on strengthening Village Health Sanitation and Nutrition Days (VHSNDs) to improve access to maternal health care, family planning services, and micro-nutrient supplementation.

Our results show that in Bihar, a significantly lower proportion of pregnant women sought ANC in the first trimester compared to other states. Further, socio-economic disparities in utilization of ANC services still exists as shown in earler research [22]. Women from the households with retun migrants were found to avail better ANC services. This is incontarst to our hypothesis that migrant families may be left behind in access to healthcare services. Other studies have documented that the outbreak of COVID-19 affected the pre-natal visits of women; the number of ANC visits declined, and there was a surge in the number of women who came for late ANC [23, 24]. It shows that community-based nutrition and health services delivery platforms, such as VHSND meetings led by community health workers, helped ensure that primary antenatal care continued to be provided to pregnant women during the pandemic and in taking the services to women's homes. The differences observed among the three states could be attributed to restriction of movement, fear of infection, and disruption of health systems and supply chains [17].

The present study established that sensitisation, counselling, and support extended to pregnant women by existing community health workers like ASHAs, ANMs, and AWWs increased after the onset of the pandemic. About 40–60 percent of the pregnant women reported that the health workers supported them in receiving ANC during the lockdown. Government guidelines to make essential services available at door step were issued [25]. During the concurrent remote progress monitoring of the Swabhimaan programme, it was noted that these community health workers often made door-to-door visits to supply IFA tablets to pregnant women and provide them with necessary counselling.

Various global health guidelines emphasise the importance of community participation in health delivery [26]. Health workers and the government should work with communities to develop, deliver, promote, and evaluate health programmes considering the diversity and vulnerability of a given community [27]. The ANC services in the *Swabhimaan* study area witnessed improvements during the COVID-19 lockdown periods. Pregnant women who got interaction with community health workers like ASHA had better exposure to these services than those who were not a part of these community platforms. Other Indian studies related to ANC and PNC service delivery during this period show that the services were disrupted due to the overburdening of the local health system due to the pandemic [16, 28–30]. As mentioned before, despite the pandemic, CHWs continued to provide services in the *Swabhimaan* study areas while also ensuring patient safety by following the guidelines of the global health agencies [31].

Our findings confirm that the ground-level community and health systems were active during the pandemic. Policies and interventions should focus on effectively using ground-level health workers to create a source of health information and access to health services, thereby reducing the impact of health emergencies in the future. Increasing the community health workforce, especially in underserved resource-poor communities, can meet the urgent demand to educate and connect people to healthcare services during emergencies. In developing countries like India, participatory community programmes such as VHSND serve as promising platforms for delivering maternal health care with the help of ASHAs, ANMs, and AWWs.

The major strength of this study is that it addresses the gap in the utilisation of ANC services before and after the pandemic based on women who were pregnant at the time of the midline and endline surveys, thus addressing the recall bias. However, it should be noted that the study was limited to selected areas. An earlier study pointed out that measuring community participation and social involvement factors will be essential to more adequately and accurately [26].

## Implication

The study highlights the importance of Community Health Workers in providing maternal and child healthcare services during the pandemic.

## Limitations

This study is not a follow-up of women from the baseline to endline. All three rounds of cross-sectional surveys were representative samples of the community. Hence, an attempt has been made to show the overall impact of CHW on ANC services. These findings are applicable to the rural areas of poor resourse setting communities in India. Further, the study could not see the impact of COVID-19 pandemic over time due to non-availability representative data over time.

## Conclusion

This study invites policymakers' attention to the new healthcare service delivery culture that supports community participation to link as many people to community health workers as possible since these platforms can potentially promote last-mile delivery of health and nutrition services in the community. The present findings suggest the positive outcomes of antenatal care supported by the CHWs during pandemic, a perfect example of community engagement that build health system resilence for health emergency. Their role in pregnancy registration, motivating for seeking ANC and distributing IFA and calcium tablets are noticeable. Women from households with return migrants did not show any bareer in access to ANC. However, the socio-economic gaps in utilization of ANC services still exist which require further attention to women from lower socio-economic starta. Furthermore, the existing ground-level community health workers network should be strengthened to ensure coproduction. These measures may form a new institutional culture to ensure the continuity of health service delivery during a pandemic, minimise the impact of a health emergency, and build resilience.

## Data Availability

The datasets used and/or analyzed during the current study are not publiclyavailable due to dissemination process at State level, but are available from Corresponding Author, on reasonable request.

## Abbreviations

ANC: Antenatal Care
ANM: Auxiliary Nursing Midwifery
ANOVA: Analysis of Variance
ASHA: Accredited Social Health Activist
AWW: Anganwadi Worker
CHW: Community Health Worker
COVID: Cornovirus Disease
IFA: Iron Folic Acid
LMIC: Low and Middle Income Countries
MU2: Mother of Children under two years
NHM: National Health Mission
PHC: Primary Health Centre
PNC: Post-Natal Care
PSM: Propensity Score Matching
PW: Pregnant Women
SARS: Severe Acute Respiratory Syndrome
SC/ST: Schedule Caste/ Schedule Tribe
TT: Tetanus toxoid
VHSND: Village Health Sanitation and Nutrition Days
WHO: World Health Organization
